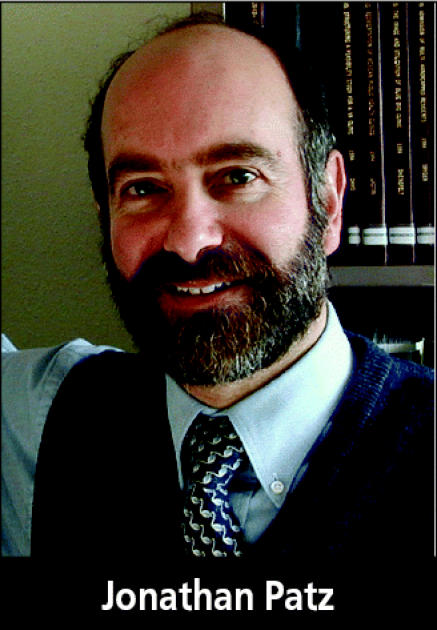# Guest Editorial: Satellite Remote Sensing Can Improve Chances of Achieving Sustainable Health

**DOI:** 10.1289/ehp.113-a84

**Published:** 2005-02

**Authors:** Jonathan Patz

**Affiliations:** University of Wisconsin–Madison, Madison, Wisconsin, E-mail: patz@wisc.edu

The Global Earth Observation System of Systems (GEOSS) is a welcomed cooperative in an era when we are becoming “data rich but knowledge poor.” With the proliferation of satellite platforms, each monitoring different characteristics of the earth’s surface and atmosphere at varying resolutions, the task of using a combination of satellite databases has been intimidating and often not possible without large analytical effort. Also, one of the most pressing challenges across the field of environmental health is obtaining accurate exposure assessments. A system that can help integrate, for instance, meteorological, air, and water pollution and soil and food contamination will improve risk assessment. Remotely sensed data are especially useful in monitoring changes in broad area or earth system disturbances; two that are especially pertinent to disease emergence include global climate change and land use change.

Many diseases or health outcomes are sensitive to climatic conditions, from mortality and morbidity due to extreme heat, cold, drought, or storms, to vector- or waterborne infectious diseases. One clear application for remotely observed data in climate-health studies is that of thermal mapping with high resolution thermal infrared aircraft (Lillesand et al. 2004). Looking at urban sprawl in aggregate, Kalnay and Cai (2003) estimated a mean surface warming due to urban sprawl and land-use change to be 0.27°C (0.49°F) for the continental United States. Thermal imagery has been combined with Landsat Thematic Mapper data in many cities; one example, Dallas, Texas, shows an urban heat island effect of 5–11°C compared to surrounding rural areas (Aniello et al. 1995). A shocking 22,000–35,000 heat-related deaths occurred across Europe during two weeks in August 2003 (IFRC 2004); if we are building cities that can raise temperatures by several degrees, we are certainly not helping future situations under climate change scenarios.

Infectious disease epidemics occur at a local or sometimes regional scale, and one key challenge to accurate vulnerability analysis is incorporating land use change projections with future projections of global climate change. For example, Hurricane Mitch, a devastating storm that hit Central America in 1998, demonstrates the combined effects of land use and extreme weather: 9,600 people perished, widespread illness from water-and vector-borne diseases ensued, and 1 million people were left homeless. Areas with extensive deforestation, with settlements on degraded hillsides or floodplains, suffered the greatest morbidity and mortality (Cockburn et al. 1999). The importance of land-cover features as a buffer to severe floods emerged as essential to long-term prevention of injuries and fatalities from floods (Glantz and Jamieson 2000).

Increasingly in recent years, meteorological satellite data has been used to model the spatial and seasonal dynamics of infectious disease transmission and develop affordable early warning systems for malaria (Thomson et al. 1997). Other climate-sensitive disease studies have combined climate and land use data to develop predictive models. Using Landsat Thematic Mapper satellite imagery, Glass et al. (2000) found that El Niño/Southern Oscillation (ENSO)-related heavy rainfall, with subsequent increase in the rodent population, preceded human cases of hantavirus pulmonary syndrome in the American southwest. And in the Bay of Bengal, Colwell and colleagues (Colwell 1996; Lobitz et al. 2000) were able to predict cholera epidemics by using AVHRR (Advanced Very High Resolution Radiometer), TOPEX/Poseidon (TOPography EXperiment for Ocean Circulation), and SeaWiFS (Sea-viewing Wide Field-of-view Sensor) remotely sensed data to determine sea-surface temperature and turbidity, sea-surface altitude, and marine algal blooms, respectively.

Using Pacific and Indian Ocean sea-surface temperature anomalies, coupled with satellite normalized difference vegetation index data, Linthicum et al. (1999) found that Rift Valley fever outbreaks could be predicted up to 5 months in advance of outbreaks in East Africa. One limitation to the use of remote sensing for the study of vector-borne disease epidemics has been cloud cover during the most critical period key to transmission for some diseases—the rainy season. Now, with the arrival of the synthetic aperture radar (SAR) that can penetrate through clouds, this problem is being resolved.

Land-use practices have had many positive impacts on human health, largely by increasing food supply, shelter, and sanitation. Nevertheless, land-use practices have also led to unintended health consequences. Road and dam construction, irrigation, habitat fragmentation, and urban sprawl all modify the transmission of infectious disease (Patz et al. 2004). Irrigation in the tropics increases the habitat and breeding sites for schistosomiasis and malaria. Dam construction has led to proliferation of the mosquito *Culex pipiens* and subsequent filariasis, or elephantiasis, near the Aswan High Dam in the southern Nile Delta (Thompson et al. 1996).

The biodiversity monitoring of GEOSS is also relevant to human health: an estimated 75% of human diseases are zoonotic, having links to either wildlife or domestic animals (Taylor et al. 2001). Lyme disease is one example of a disease linked to forest fragmentation in the eastern United States, with subsequent proliferation of deer and white-footed mice key in the pathogen’s life cycle. Combining remotely sensed land use data with statistical software to analyze habitat fragmentation patterns could, therefore, potentially enhance Lyme disease risk predictions.

Finally, lessons for building resilience against unpredictable catastrophes are emerging from the recent tragic tsunami in the Indian Ocean that, at last report, has killed upwards of 150,000 people, with many more injured or at risk of infectious diseases. Improved satellite early warning systems are already under discussion, but additional evidence is emerging about high survival rates of people in areas with intact coral reefs and mangroves. These types of land use change are best studied with satellite remote sensing in combination with local ground-truth data.

In summary, the goals of GEOSS’ 10-year international collaboration to greatly improve data compatibility and communication across earth-observing systems has particular relevance to human health. The goals of disaster reduction, water resource management, ocean and marine resource management, air-quality monitoring, biodiversity monitoring, and sustainable land use management could not be more central to understanding human population vulnerability across the generations.

## Figures and Tables

**Figure:**